# Iterative finite element analysis of molar uprighting using superelastic Nickel Titanium spring: introducing an Element-Iteration-Specific (EIS) material model

**DOI:** 10.1186/s40510-026-00636-z

**Published:** 2026-06-29

**Authors:** Lama A. AlKahlan, Abdelhafid M. Mallek, Mohamed Z. Bendjaballah, Naif A. Bindayel

**Affiliations:** 1 Department of Pediatric Dentistry and Orthodontics, Division of Orthodontics College of Dentistry, King Saud University, Riyadh, Saudi Arabia; 2 Laboratory of Mechanics, Physics and Materials (LMPM), Department of Mechanical Engineering, University of Sidi Bel Abbes, Sidi Bel Abbes, Algeria; 3 Biomedical Technology Department, College of Applied Medical Sciences, King Saud University, Riyadh, Saudi Arabia

**Keywords:** Mesially tilted molar, Uprighting spring, Superelastic nickel titanium, Material modeling, Orthodontic biomechanics, Iterative finite element analysis

## Abstract

**Background:**

Superelastic Nickel Titanium (NiTi) exhibits nonlinear and path-dependent behavior that complicates accurate simulation of orthodontic appliances. Standard iterative finite element (FE) formulations often fail to maintain realistic stress evolution during simulations, leading to non-physiologic force predictions. This study introduced an Element-Iteration-Specific (EIS) material model implemented within the FE framework to incorporate the behavior of superelastic NiTi alloy into an iterative simulation, aiming to investigate the biomechanics of a molar uprighting spring and estimate the clinical treatment duration.

**Methods:**

A two-dimensional (2D) model was constructed representing a 30° mesially tilted mandibular second molar uprighted using a prefabricated superelastic NiTi spring. At each iteration, the EIS algorithm redefines the element specific material parameters of the spring according to the stress–strain state inherited from the preceding iteration, thereby preserving the continuity of its behavior and enabling realistic stress evolution and load transfer between the wire, brackets, and teeth.

**Results:**

The simulation achieved 23.3° of molar uprighting with 3.0 mm of tangential distal and 2.4 mm of normal extrusive displacements, over an equivalent of 14-week clinical duration. The NiTi spring generated an initial 12.4 N·mm (~ 1265 g·mm) counter-clockwise moment and 0.9 N normal extrusive force. The mean PDL stress remained physiologic, decreasing from 20.3 KPa at wire activation to 13.3 KPa at the end of the simulation. The model successfully tracked the spatial and temporal evolution of the superelastic NiTi’s stress-induced martensitic transformation during activation and uprighting.

**Conclusions:**

The EIS framework effectively reproduced the nonlinear and history-dependent response of superelastic NiTi, offering clinically representative predictions and establishing a validated computational foundation for optimizing NiTi-based orthodontic appliances and improving treatment outcomes.

## Background

Mesially tilted mandibular second molars are among the frequent associated findings with missing first molars [1]. Such angled molar positions occupy the adjacent space, create limitations for prosthodontic treatment, and often present with signs of deteriorating periodontal health, including inflammations, bone resorption, and pseudopockets [2]. To achieve a functional occlusion and prevent anatomical disturbances, the treatment of choice for such cases is either orthodontic space closure or prosthetic replacement [3]. When the space for a pontic is compromised, the mesial side of a tilted molar is at risk of overpreparation to provide an acceptable path of insertion for the fixed prosthesis. This may expose the pulp and subject the tooth to improper loading. Therefore, orthodontic molar uprighting is often the preferred treatment for preserving and enhancing the periodontal environment in the region while creating appropriate space for a missing first molar’s prosthetic replacement [4].

Molar uprighting can be performed using a variety of appliances [1, 2, 5]. Among the first to present such an appliance was Burstone, who in 1966 introduced a conventional molar uprighting spring consisting of a manually shaped rigid stainless steel (SS) wire segment designed to engage the molar. This traditional approach remains effective but depends heavily on operator skill and requires frequent reactivation [6, 7].

As new advances emerged in the field of orthodontics, more materials have been incorporated into molar uprighting treatment. A prominent example is superelastic Nickel Titanium (NiTi) alloy, characterized by high elasticity and shape memory, which allows for continuous light force delivery and makes it preferable to SS in certain clinical situations [5, 8–10]. The two unique aspects of such alloy material are its stress-induced superelastic behavior and its temperature-induced thermoelastic behavior [11]. Superelasticity arises from reversible martensitic phase transformations. During bracket engagement, the archwire undergoes an austenitic-to-martensitic transformation as it deflects, increasing its flexibility, and then returns to the austenitic phase to regain its original shape, thereby delivering nearly constant forces even at considerable deflections [12–14]. Thermoelasticity, on the other hand, is expressed when insertion of the archwire into the oral cavity raises its temperature, triggering a martensitic-to-austenitic transformation that stiffens the wire, increases force delivery, and enhances torque expression and tooth movement [15, 16].

These distinctive material properties have encouraged the development of orthodontic utilities designed to exploit superelastic NiTi for greater treatment efficiency. Among the introduced molar uprighting appliances utilizing such material is the prefabricated Memory Titanol^®^ spring (Forestadent, Bernard Förster GmbH, Pforzheim, Germany). This spring provides flexibility during molar engagement, which is especially useful when access is difficult due to the tilted position. Its shape memory properties eliminate the need for frequent reactivations while delivering constant forces to achieve the desired uprighting, offering convenience for both clinician and patient [2, 17, 18].

To better understand and optimize the biomechanics of such appliances, finite element (FE) analysis has become one of the most important numerical tools in dental research [19–21]. FE analysis enables detailed evaluation of the force effects of orthodontic appliances, including their impact on the periodontal ligament (PDL) and resulting tooth movements [22]. Additionally, it allows for the detailed evaluation of mechanical properties related to different dental materials [23, 24]. However, molar uprighting springs have been investigated in only a limited number of studies [25–28].

Previous FE studies on long-term orthodontic simulations have taken different directions. Some incorporated explicit bone remodeling formulations, applying mechanobiological laws such as density adaptation [29], strain energy density [30], or a strain-based bone remodeling formulation applied in conjunction with superelastic NiTi mechanics [31], to reproduce resorption and apposition of alveolar bone over time. In the latter study, the superelastic material properties of NiTi were implemented via a UMAT user subroutine, with the bone remodeling rate defined as a function of strain in the PDL. Each computational cycle of loading and remodeling was assumed to correspond to one biological day, an assignment inherent to the model formulation rather than supported by clinical calibration. Others emphasized validation against clinical imaging, using computed tomography (CT) or intraoral scans to confirm the accuracy of tooth displacement predictions without embedding long-term remodeling mechanisms [32, 33].

Iterative FE approaches with geometric updates have also been developed, in which explicit biological processes of bone remodeling are discarded and tooth displacement is simulated by repeatedly activating the appliance and updating the loading conditions and geometry until the desired correction is achieved. In this framework, the geometry of the PDLs and surrounding alveolar bone is continuously adjusted to represent the cumulative mechanical effect of PDL deformation and the remodeling driven changes observed clinically. To date, however, only a limited number of studies have employed this approach, and although such models provide a practical approximation of long-term movement, they lack a clinically calibrated correlation between iteration count and treatment duration [34, 35].

Only recently has the estimation of clinical treatment duration through iterative FE analysis become feasible, as demonstrated by the work of AlKahlan et al. (2025) [36]. In that study, extensive iterative FE simulation of molar uprighting was performed. Iteration duration was deduced from experimental and clinical canine retraction studies by establishing a relationship between average mean stress in the PDL and bone remodeling velocity. At each step, the normal displacement of the PDL surface was divided by the corresponding remodeling velocity to calculate its real-time equivalent. Summation of all iteration durations then yielded, for the first time, a reliable prediction of overall clinical treatment duration. This novel method bridged the gap between computational biomechanics and clinical orthodontics by enabling treatment time estimation directly from iterative FE analysis.

When conventional materials such as SS are used, iterative modeling is relatively straightforward, as each deformation state corresponds to a unique and predictable stress response, owing to the linear and history-independent behavior of the material [26, 37, 38]. However, when superelastic NiTi is incorporated, the modeling becomes significantly more complex, due to the alloy’s nonlinear and path-dependent properties, where a single deformation state may correspond to multiple stress states depending on whether the material is loading or unloading [31, 39]. If these complexities remain unresolved, stress–strain mismatches accumulate over consecutive iterations, leading to unrealistic simulation predictions or even convergence failure [13, 40]. This challenge accentuates the need for modeling strategies capable of updating material properties across successive iterations for each position along the orthodontic wire, ensuring the correct stress state is maintained throughout the simulation.

Thus, the primary objective of this study was to introduce an Element-Iteration-Specific (EIS) model and apply it together with the previously developed iteration-to-clinical duration estimation method [36], in order to (i) investigate the kinetic and kinematic responses of a mesially tilted mandibular second molar uprighted with a superelastic NiTi spring, (ii) analyze the alloy’s superelastic behavior relating to the reversible stress-induced austenitic to martensitic transformation during stepwise deformation recovery, and (iii) estimate the corresponding orthodontic treatment duration using iterative FE analysis. The null hypothesis of this study was that the EIS model incorporated into the iterative simulation of molar uprighting would not produce clinically realistic predictions of tooth movements, superelastic NiTi behavior, and treatment duration.

## Methods

### Finite element model generation

SolidWorks^®^ software (Version 2022, Dassault Systèmes, Vélizy-Villacoublay, France), was used to create a two-dimensional (2D) computer-assisted design (CAD) model to simulate the molar uprighting process. The first step involved reconstructing a three-dimensional (3D) model of the mandibular right posterior dental segment from a CT scan image that was sourced from osirix database’s DICOM-image repository [41]. The edges of the 3D CAD model were then extracted along a central plane that intersected the middle of the teeth, transforming it into a 2D CAD representation. The PDL was modeled with a uniform thickness of 0.2 mm, a value broadly acknowledged by previous literature [26, 42]. While this uniform assumption does not capture local variations such as root concavities or patient specific irregularities, it provides a robust and consistent basis for predicting the overall biomechanics of molar uprighting within the iterative simulation process. Similarly, the dental geometry was simplified to preserve key morphological features relevant to sagittal plane movement, while omitting finer anatomical details. These modeling choices were intentional, prioritizing the accurate capture of the overall biomechanical patterns governing molar uprighting over resolving localized stress concentrations. It also ensures sufficient reliability for the proposed method designed to overcome the challenges associated with superelastic NiTi material in FE iterative analyses.

The model’s specifications and constituents were adapted from a previous FE study [36]. These include a 30° tilted mandibular second molar and an anchorage teeth segment consisting of mandibular canine, first and second premolars with surrounding alveolar bone. The mandibular first molar was omitted to reproduce an edentulous first-molar space, where uprighting of the mesially tilted second molar is clinically performed to regain prosthetic space for implant insertion. The appliances included are: 0.022-inch slot size standard edgewise brackets bonded to the anchorage teeth, an edgewise molar buccal tube with 4 mm width, a segmental 0.019 × 0.025-inch SS wire linking the anchorage teeth, and a prefabricated molar uprighting Memory Titanol^®^ spring (Forestadent, Bernard Förster GmbH, Pforzheim, Germany).

**Fig. 1:**
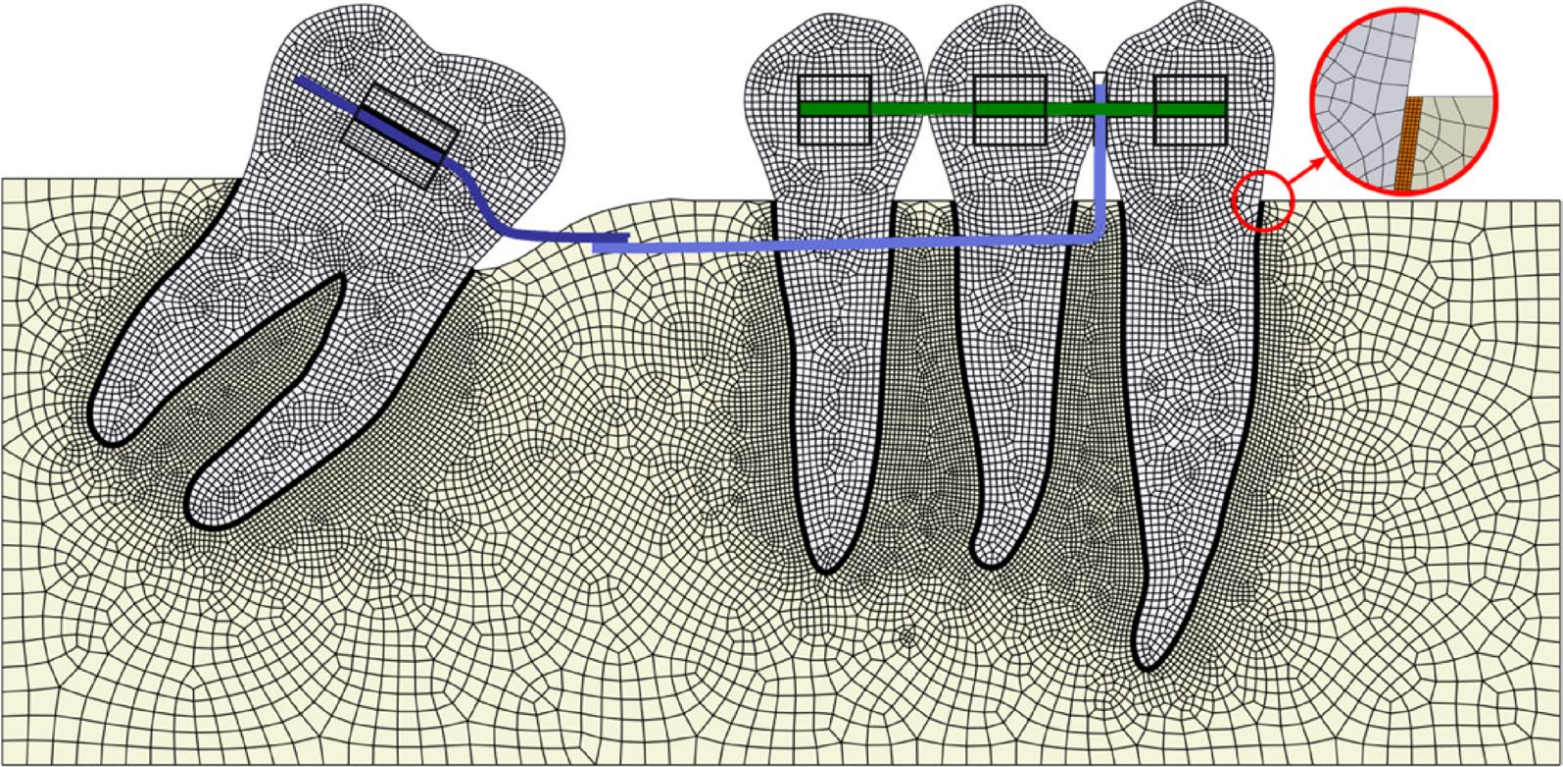
Finite element (FE) model of the molar uprighting spring. The FE mesh is shown following the activation of the superelastic Nickel Titanium (NiTi) and stainless steel (SS) spring components. The model includes a mesially tilted mandibular second molar with a buccal tube, anchorage teeth with brackets, the periodontal ligament (PDL), and the surrounding alveolar bone. The NiTi and SS wire components were discretized using B21 beam elements. Owing to the one-dimensional nature of these elements and the very fine mesh employed in contact regions, the individual beam nodes and element boundaries are not readily discernible in the figure

The molar uprighting spring consisted of two sections rigidly joined by a crimpable tube. The first section had an L-shaped 0.017 × 0.022-inch SS wire that is connected to the anchorage wire via a cross tube positioned between the canine and first premolar. The second section was made of a 0.016 × 0.022-inch superelastic NiTi wire with a preactivated 30° bend engaging the tilted molar’s buccal tube (Fig. 1). In this arrangement, the SS section provided stable anchorage, while the activated NiTi section delivered the uprighting moment to the molar [2].

As shown in Fig. 1, a 2D plane stress mesh was created by Abaqus/Standard software (Version 2022, Dassault Systèmes, Vélizy-Villacoublay, France), using reduced integrated linear quadrilateral elements (CPS4R). Two-node linear beam elements (B21) were used to model all wire components of the uprighting system, including the superelastic NiTi wire, the SS wire segment of the uprighting spring, and the SS anchorage wire. These components were discretized using 152, 127, and 323 elements, respectively. The wire portions located within the molar buccal tube, cross tube, and anchorage brackets were meshed with a fine B21 element size of 0.05 mm to better capture the load transfer between the wires and orthodontic appliances. Furthermore, to enhance accuracy in regions with high stress gradients, the mesh was also locally refined around the PDL and root surfaces. A Medial Axis Algorithm was used for mesh control to enable symmetrical distribution and displacement transmission along the same normal vector for elements in contact around the PDL–bone and PDL-root interfaces. A preliminary mesh sensitivity analysis was performed using progressively finer meshes to determine an appropriate element size. The solution was considered converged at an element size of 0.05 mm, as displacement and stress values varied by less than 2% between successive refinements. Since at least four elements were assigned across the PDL thickness, the hourglass effects inherent to reduced integration elements were substantially minimized [43]. During the first iteration, the PDLs of the second molar, second premolar, first premolar, and canine were discretized with 4,279, 2,496, 2,488, and 3,148 CPS4R elements, respectively. In addition, the bone was refined with an element size of 0.2 mm at the PDL–bone interfaces and gradually coarsened toward the outer boundaries, resulting in up to 10,123 elements within the bone domain.

Regarding the boundary and contact conditions, the mesial and distal borders of the alveolar bone domain were constrained to prevent rigid-body motion. These boundaries were selected to reduce computational cost while maintaining mechanical stability as the model showed truncation at these locations. Tie contact was assumed between the PDL–bone and PDL–root interfaces. A Beam Multi-Point Constraint (MPC) was applied between the tips of the superelastic NiTi and SS wires to simulate their rigid attachment. Frictional surface-to-surface contacts were defined between the NiTi wire and the buccal tube, and between the SS anchorage wire and the brackets, each with a friction coefficient of 0.28, consistent with values reported in previous studies [44–47].

Using Abaqus/Standard software to carry out the molar uprighting treatment simulation, the spring was first activated by inserting the L-shaped section of the SS wire into the cross tube and contouring the exposed arm of the superelastic NiTi wire by 50° to engage the molar’s buccal tube. Upon spring release, the contact forces between the molar buccal tube, brackets and uprighting spring, together with the resulting PDL deformations, drove tooth movements until equilibrium was achieved. A Python 2.7 script was integrated into the workflow after the first iteration to automate successive runs and manage geometric updates.

In each iteration, the script reinserted all components and applied mating conditions to reposition the teeth according to their previous locations, and reconstructed the PDLs and bone through Boolean operations. The PDLs were set to have a 0.2 mm uniform thickness to represent alveolar bone remodeling. The meshing procedure was again performed, and prescribed displacements were imposed on the superelastic NiTi and SS wire nodes to restore their positions attained at the end of the previous iteration within the molar’s buccal tube, the brackets, and the cross tube. The script included an internal loop that executed these steps sequentially and automatically launched each simulation. The underlying premise of this simulation was that the initial tooth displacement dictates subsequent orthodontic tooth movement [48], a concept previously implemented in FE simulations of long-term orthodontic movement [35].

**Fig. 2:**
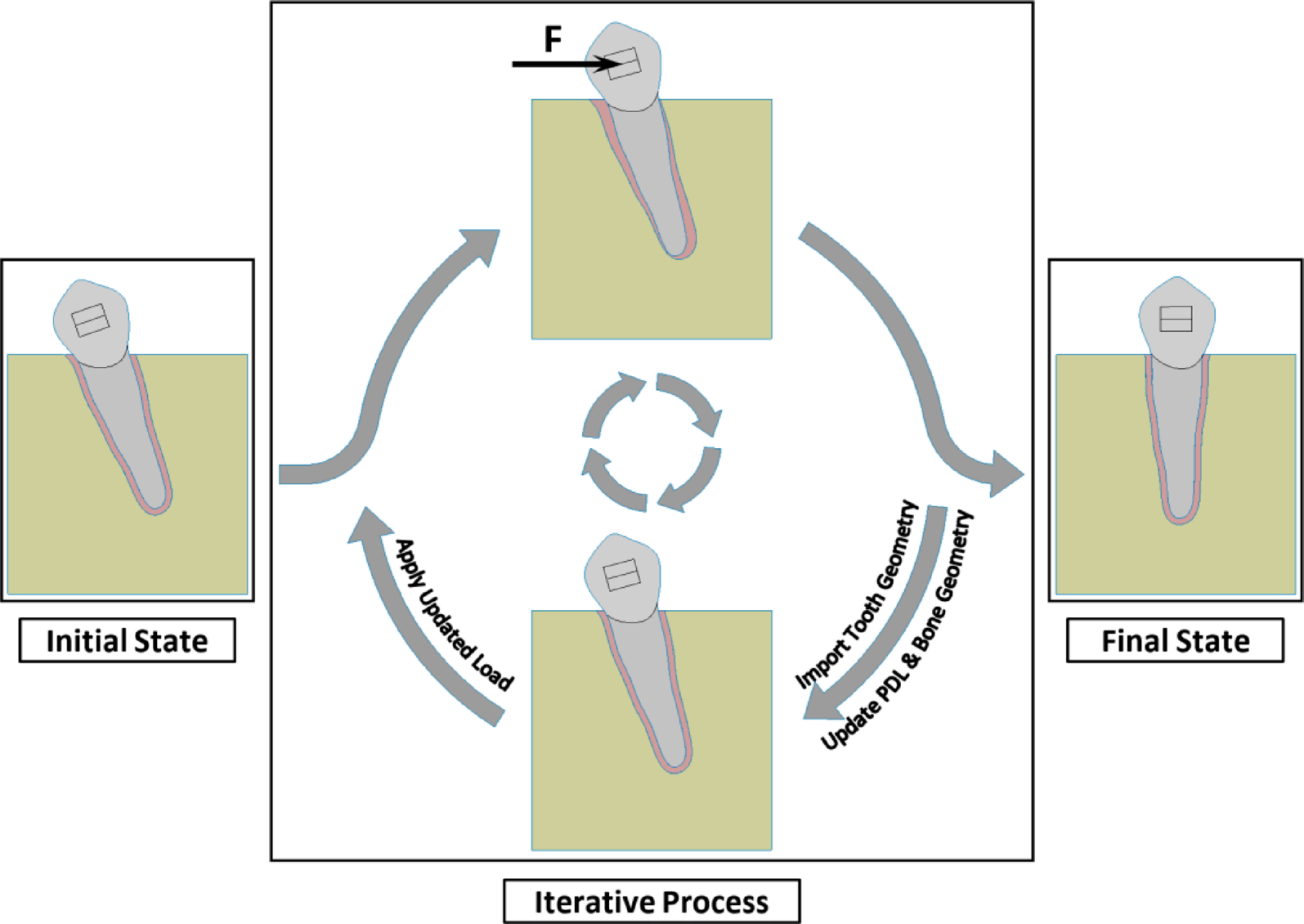
The iterative process for simulating long-term orthodontic tooth movement. At each iteration, the applied force (*F)* produces PDL deformation, which drives alveolar bone remodeling and is reflected in the updated geometry. This cycle continues until the required orthodontic correction is achieved or the corrective forces dissipate

During the iterative process, the intermediate biological transitions were omitted. Instead, the model geometry was directly updated to represent the post-remodeling configuration before solving for a new equilibrium state at the next iteration. The iterative cycle used to approximate long-term orthodontic tooth movement is illustrated in Fig. 2 using a simplified canine model for clarity. At each iteration, the applied force produced PDL deformation, after which the bone–PDL–tooth geometry was updated to represent the cumulative remodeling outcome at that step. The updated geometry then served as the starting configuration for the next FE iteration, where the loading conditions reached at the end of the previous step were reapplied and a new equilibrium state was calculated. This cycle was repeated until the required orthodontic correction was achieved or the corrective forces had dissipated, yielding the final equilibrium state.

The clinical treatment duration was estimated using a previously established novel FE method [36]. The simulation outcomes, including tooth displacements and PDL deformations, were used to calculate the real-time duration of each iteration in the molar uprighting process when using the superelastic NiTi spring. This process relates the applied orthodontic forces to the PDL stress levels and subsequent displacement, which is translated into bone remodeling velocities, according to a previously established correlation with clinical observations and experimental data. Using this correlation, the equivalent clinical duration was extracted for each numerical step, where at a given iteration, applied forces would dictate the rate of tooth movement in a real-time manner.

### Material properties

Table 1 shows the mechanical properties of biological structures and SS alloy involved in the FE model obtained from previous literature [31]. Every component was regarded as linear, elastic, homogeneous, and isotropic, with the exception of the molar buccal tube, cross tube, and brackets, which were regarded as rigid materials.

**Table 1:** Mechanical properties of the structures involved in the finite element (FE) model

Material	Young’s modulus (MPa)	Poisson’s ratio (ν)
Bone	3,000	0.3
PDL	0.84	0.46
Teeth (Root/Dentin)	20,000	0.3
Teeth (Crown/Enamel)	70,000	0.3
Stainless Steel	200,000	0.3

The superelastic NiTi alloy used in the active segment of the spring responsible for transmitting the uprighting moment to the molar was selected for its distinctive properties, which allow it to deliver nearly constant forces across a wide range of deformations. This superelastic behavior arises from the stress-induced martensitic transformation, permitting such wires to undergo substantial reversible strains without permanent deformation. In the FE model, the superelastic NiTi material was defined in Abaqus/Standard software using native parameters that describe the boundaries and slopes of its transformation behavior: the austenite and martensite Young’s moduli (*E*_*A*_ and *E*_*M*_), the start and end transformation stresses during loading (*STL* and *ETL* respectively), the start and end transformation stresses during unloading (*STU* and *ETU* respectively), and the transformation strain (*TS*). For accurate simulation in both tension and compression, the start transformation stress in compression (*STL_C*) is also required by all major FE solvers. Together, these parameters govern the transition between austenitic and martensitic phases under both tension and compression, ensuring the simulated response faithfully captures the nonlinear, path-dependent behavior of superelastic NiTi in orthodontic applications. Table 2 presents the specific values used in this study adopted from previous literature [9, 12].

**Table 2:** Superelastic Nickel Titanium (NiTi) material parameters used in the FE simulation model

$\:{\boldsymbol{\sigma\:}}_{\boldsymbol{S}\boldsymbol{T}\boldsymbol{L}}$	$\:{\boldsymbol{\sigma\:}}_{\boldsymbol{E}\boldsymbol{T}\boldsymbol{L}}$	$\:{\boldsymbol{\sigma\:}}_{\boldsymbol{S}\boldsymbol{T}\boldsymbol{U}}$	$\:{\boldsymbol{\sigma\:}}_{\boldsymbol{E}\boldsymbol{T}\boldsymbol{U}}$	$\:\boldsymbol{T}\boldsymbol{S}$	$\:{\boldsymbol{E}}_{\boldsymbol{A}}$	$\:{\boldsymbol{E}}_{\boldsymbol{M}}$	$\:{\boldsymbol{\sigma\:}}_{\boldsymbol{S}\boldsymbol{T}\boldsymbol{L}\_\boldsymbol{C}}$
377 MPa	430 MPa	200 MPa	160 MPa	5.2%	44 GPa	23 GPa	452 MPa

*STL* Start transformation stress in loading. *ETL* End transformation stress in loading. *STU* Start transformation stress in unloading. *ETU* End transformation stress in unloading. *TS* Transformation strain. *E*_*A*_ Austenite elastic moduli. *E*_*M*_ Martensite elastic moduli. *STL_C* Start of transformation in compression loading

**Fig. 3:**
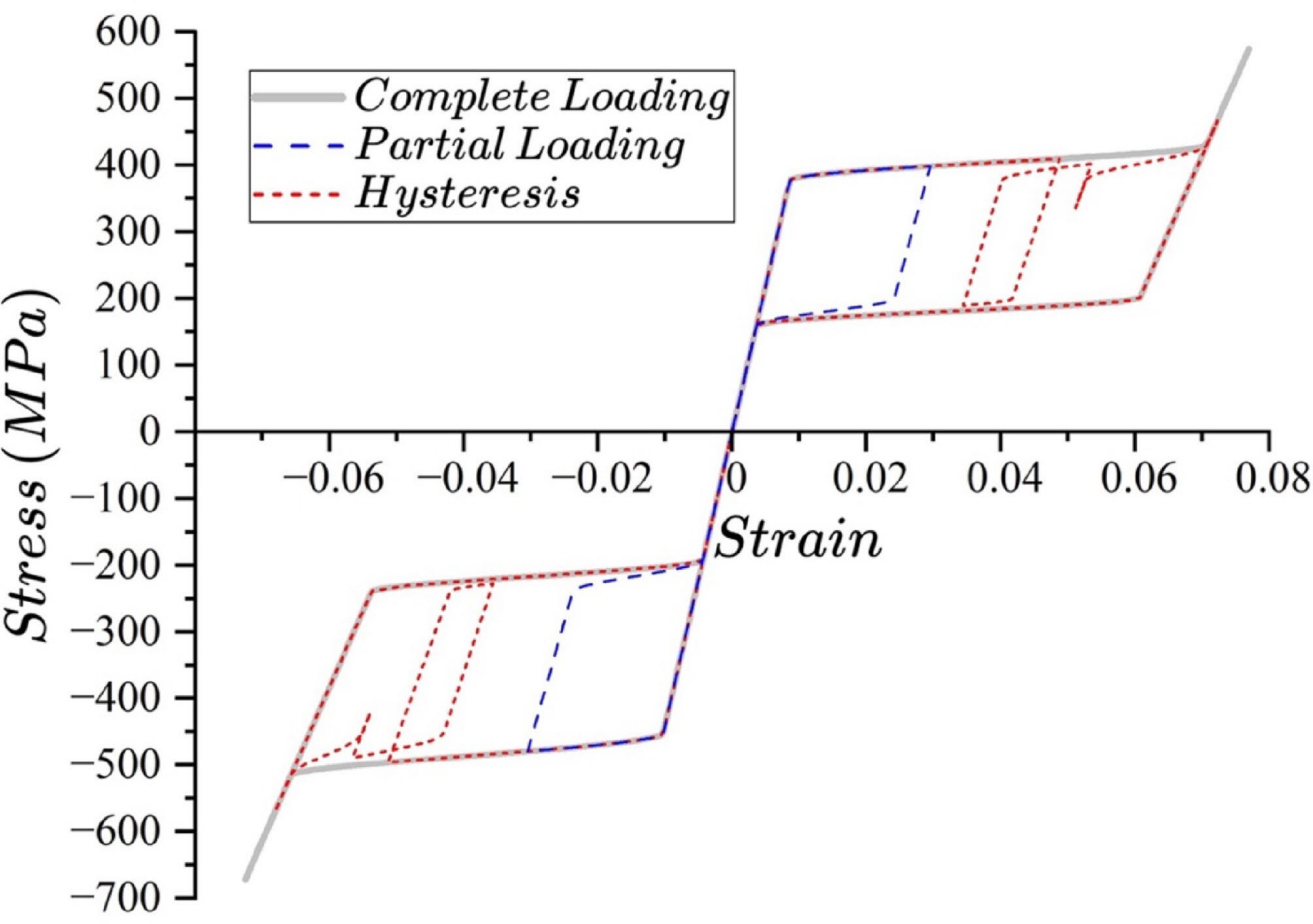
Simulated stress–strain response of superelastic NiTi under pure tensile and compressive uniaxial loading tests. Positive values illustrate the transformation behavior in tension, and negative values illustrate the transformation behavior in compression. The complete loading test is shown by the solid gray line, the partial loading test by the blue dashed line, and the hysteresis response by the red dashed line

To illustrate and validate the modeled stress–strain response of superelastic NiTi, pure tensile and compressive uniaxial loading tests were incorporated in the FE analysis [9, 12]. In the first test, the applied load was sufficiently high for the tensile and compressive stresses in a selected element to exceed the respective end transformation stresses in loading ETL, after which the specimen was fully unloaded. In the second test, the induced stresses were between the start STL and end ETL transformation stresses in loading, followed by complete unloading. In the third test, the induced stresses were also between STL and ETL, but the wire was only partially unloaded before being reloaded twice, enabling observation of the material’s hysteresis behavior. The stress–strain curves obtained from these tests are presented in Fig. 3.

To ensure accurate reproduction of both deformation and force histories during the simulation, particular attention was given to how superelastic NiTi behavior was handled between iterations. During the iterative simulation, reapplying the nodal displacements from the previous iteration restores the deformation state, but may not reproduce the correct stress state because of the path-dependent superelastic response. Unlike conventional elastic materials, in which a given strain corresponds to a unique stress, superelastic NiTi may exhibit different stress levels at the same strain depending on the preceding loading or unloading path [13, 14, 39].

**Fig. 4:**
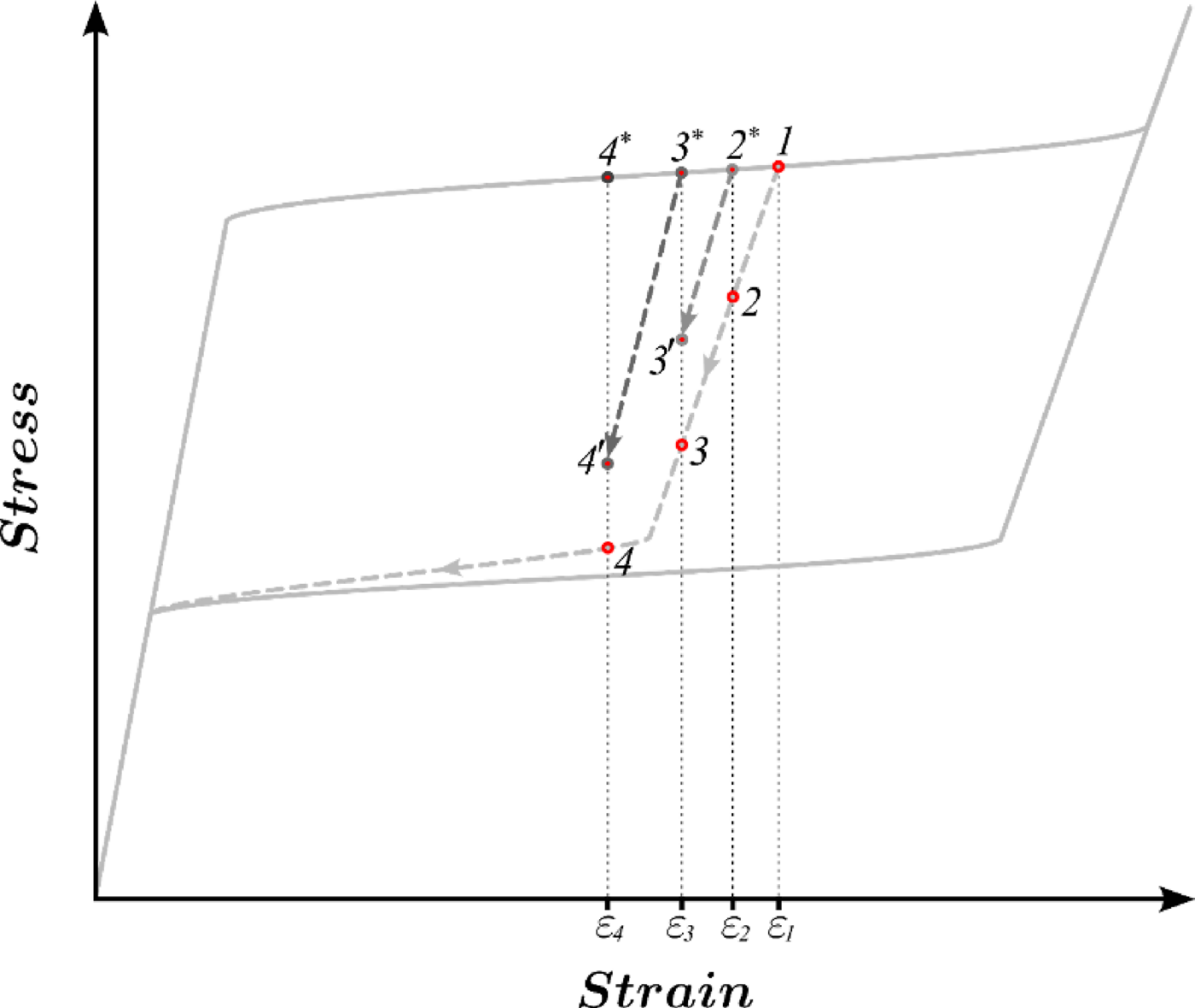
Path-dependence problem in superelastic NiTi during iterative FE analysis. Strain ε_2_ is assigned to the loading branch 2* instead of the true unloading state 2, causing subsequent unloading to follow 2*–3’ rather than the sought 2–3 path, leading to progressive divergence from the actual material response

This problem is illustrated in Fig. 4. When a superelastic NiTi element is initially activated up to a strain ε₁ and subsequently released within the appliance constraints, the unloading response follows its unloading trajectory until mechanical equilibrium is reached between the molar and the surrounding structures, corresponding to a residual strain ε₂. If the original constitutive properties of the material are directly reapplied in the following iteration, ε₂ is interpreted as state 2*, associated with a stress level markedly higher than the true terminal state 2 reached at the end of the preceding iteration. As unloading continues, the trajectory follows 2*–3′ instead of 2–3. Over successive iterations, this discrepancy accumulates; for example, the next iteration starts at 3* instead of 3 and ends at 4′ instead of 4, leading to progressive divergence between the simulated response and the true unloading behavior of superelastic NiTi.

**Fig. 5:**
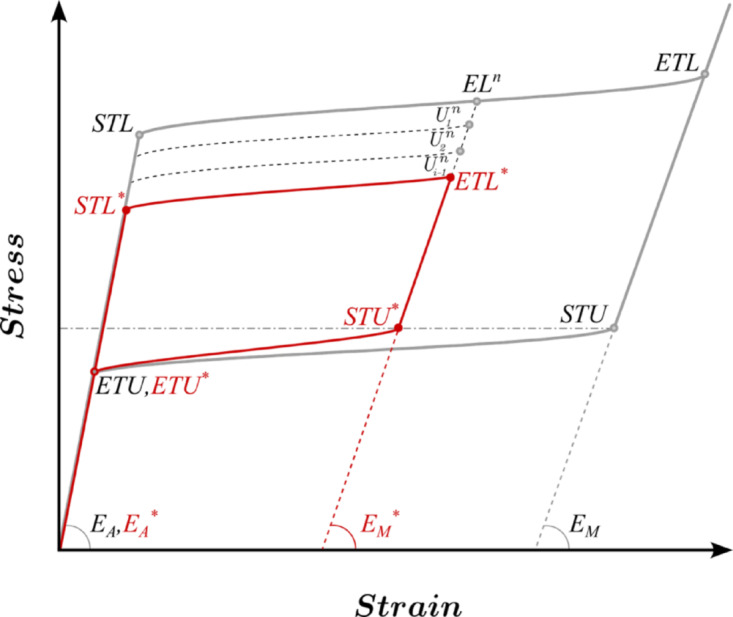
The Element-Iteration-Specific (EIS) superelastic NiTi model’s stress–strain relationship. The original material behavior can be seen represented by the grey curve. The red curve represents the EIS stress–strain response, which is a locally updated version of the original superelastic NiTi model for a specific element n at a specific iteration *i*, whose parameters are marked with an asterisk (*) and derived primarily from the unloading state at iteration $\:{U}_{i-1}^{n}$

During the implementation of the iterative process, the stress–strain state of each NiTi element was evaluated at the end of every iteration. If the element was following an unloading path, a new set of superelastic material parameters was generated and assigned to that element in the following iteration to recover the stress corresponding to the previously attained strain, as seen in Fig. 5. If the element remained on the original loading path, the original superelastic material parameters were retained, since no unloading-path correction was required.

To address this issue, we introduced an EIS model, which ensures, first, that the element is consistently returned to the stress–strain state attained at the end of the previous iteration, and second, that unloading always proceeds along the same material path. This technique redefines each element’s material parameters at every iteration based on its actual stress–strain state from the preceding iteration, ensuring continuity of both strain and stress histories. The EIS model was developed to tackle the inability of standard FE solvers to recover the correct stress state of superelastic NiTi when re-establishing a prior strain condition during the iterative simulation of orthodontic tooth movement. As shown in Fig. 5, the original material stress–strain response (grey curve) is defined by global parameters such as *E*_*A*_, *E*_*M*_, *STL*,* ETL*,* STU*,* ETU*, and *TS*. In typical solver behavior, when an element *n* at iteration *i* is reset to the strain level reached in the previous iteration ($\:{U}_{i-1}^{n}$), the solver assumes the element lies on the loading branch, disregarding whether the actual prior state was in unloading. This results in inaccurate stress assignment. To overcome this, the EIS model generates a locally updated material curve (red curve) representing the stress–strain response for each element *n* at each iteration *i.* The process begins by identifying the most recent unloading state $\:{U}_{i-1}^{n}$ from the prior iteration, which serves as the reference point for defining the element specific stress–strain loop. While *E*_A_*, STU* and ETU* remain unchanged, ETL*,* E*_M_* and TS* are extracted from the previous unloading $\:{U}_{i-1}^{n}\:$and the end of loading *EL*^*n*^ states for the element *n*. The start transformation in loading STL* is then deduced from these updated parameters to ensure internal consistency of the transformation plateau. This EIS recalibration preserves the correct phase transformation path and stress level upon reloading or further unloading, allowing the FE simulation to capture superelastic NiTi’s nonlinear, path-dependent behavior accurately throughout the molar uprighting process. The start transformation in compression STL_C* is then calculated from STL* while preserving the ratio *STL/STL_C* between the original and EIS material models, thereby maintaining the correct tensile–compressive asymmetry.

## Results

### Treatment duration and alloy stress behavior

**Fig. 6:**
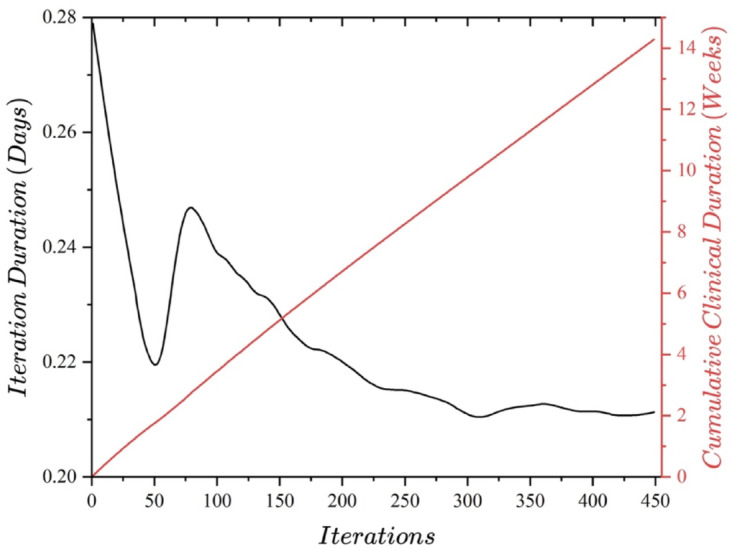
Estimated iteration duration in days and cumulative clinical duration in weeks throughout the treatment simulation

The estimated clinical duration (red curve) corresponding to each computational iteration (black curve) of the molar uprighting treatment can be seen in Fig. 6. At the beginning of the simulation, the iteration duration was longest, followed by a pronounced dip up to the 50th iteration and a mild increase until around the 75th. Beyond this point, the iteration duration gradually shortened and stabilized into an approximately uniform plateau. The entire uprighting process was completed after 450 iterations, corresponding to an equivalent of 14 weeks of cumulative clinical duration.

**Fig. 7:**
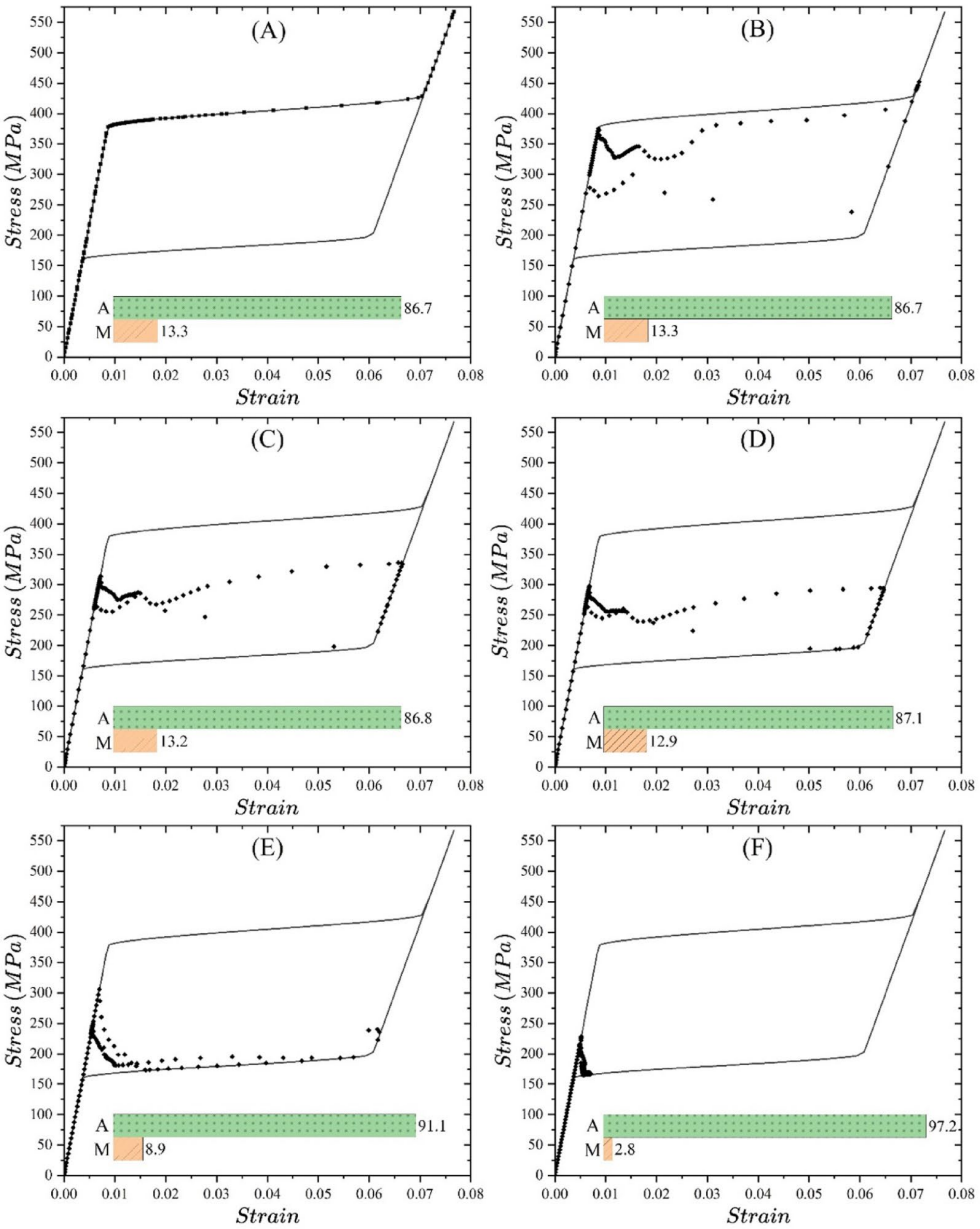
Stress behavior of the superelastic NiTi spring during molar uprighting. Normal stress in the bottom fiber of the wire elements is displayed at different stages of the iterative simulation: **A** end of wire activation, **B** end of wire release, **C** iteration 50, **D** iteration 70, **E** iteration 220, and **F** iteration 450. Each panel also displays the corresponding phase fractions, showing the percentage of martensite (orange) and austenite (green) fractions present during transformation

Figure 7 illustrates the evolution of stress and phase transformation in the superelastic NiTi spring during molar uprighting. The plots show the longitudinal normal stress in the bottom fiber of the wire, where the highest tensile stresses occur, at key stages of the simulation together with the corresponding martensite (orange) and austenite (green) fractions. The selected iterations represent the initial spring activation–release step (A and B respectively), early stages at iterations 50 and 70 where distinct changes in iteration duration were observed (C and D respectively), mid-treatment at iteration 220 when approximately half of the cumulative clinical duration had elapsed (E), and the final stage of molar correction at iteration 450 (F). These snapshots also capture the superelastic NiTi’s behavior progression in correspondence with treatment duration. During the wire activation step, the martensitic phase peaked at ~ 13.3%. Elements that were partially transformed lay on the transformation portion of the stress–strain curve, with stresses between the STL (377 MPa) and ETL (430 MPa), whereas fully transformed elements entered the elastic martensite regime, carrying stresses between ~ 430 MPa and ~ 567 MPa. As the simulation progressed, stresses gradually decreased while the alloy transformed back toward the austenitic phase. From approximately iteration 220 onward, unloading drove the elements that had previously undergone martensitic transformation into the reverse-transformation plateau, where the stress decreased only modestly from about 200 MPa to 160 MPa. The orthodontic correction then progressed steadily under this nearly constant load until the end of treatment. At completion, the alloy composition had shifted to 97.2% austenite and 2.8% martensite, indicating recovery from the stress-induced martensitic phase while maintaining residual loading through austenite deformation.

**Fig. 8:**
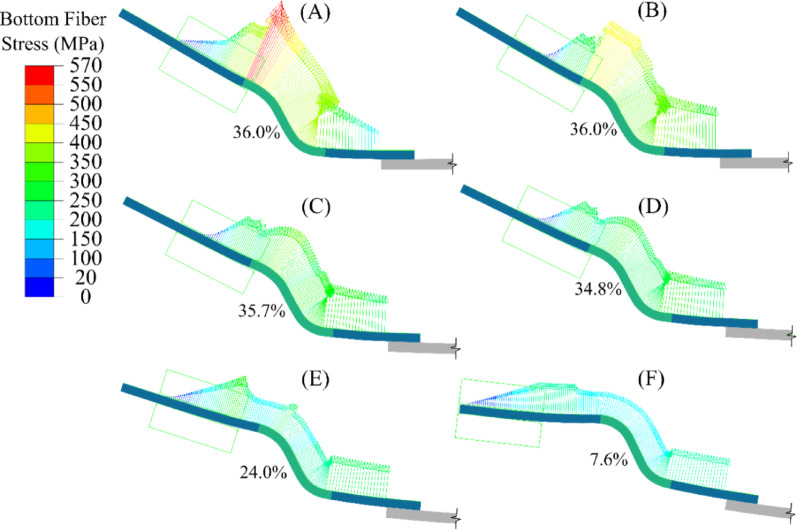
Detailed distribution of normal stress and martensitic phase percentages within the superelastic NiTi uprighting spring. The color map represents normal stress in the bottom fiber of the wire, displayed at the same representative stages in the previous figure: **A** end of wire activation, **B** end of wire release, **C** iteration 50, **D** iteration 70, **E** iteration 220, and **F** iteration 450. Annotated values indicate the local martensitic fractions within the middle segment of the wire, which is the only portion that underwent martensitic transformation

The detailed distribution of normal stress within the spring’s superelastic NiTi component is shown in Fig. 8 at the same representative stages of the iterative simulation discussed previously: (A) end of wire activation, (B) end of wire release, (C) iteration 50, (D) iteration 70, (E) iteration 220, and (F) iteration 450. The color map displays the normal stress along the bottom fiber of the wire, where tensile stresses are highest. The analysis revealed that the spring consistently maintained three distinct regions with stable length ratios of approximately 35% (left), 37% (middle), and 28% (right) throughout all stages. The left and right regions remained entirely austenitic, whereas the middle-deflected segment exhibited varying levels of stress-induced martensitic transformation. The annotated percentages in each subfigure denote the extent of martensitic phase transformation within that particular portion of the wire, from which the overall martensite levels shown in Fig. 7 can be retrieved. The largest stress-induced martensitic transformation, around 36%, was observed during the spring activation step at the highest stress value of approximately 567 MPa. As molar uprighting progressed, both stress levels and martensitic fractions gradually decreased, reflecting the alloy’s recovery. A key aspect of this recovery occurred along the reverse-transformation plateau, which began around iteration 220, equating approximately to week 7 of clinical duration, where stresses steadily decayed from about 200 MPa to 160 MPa over the remaining duration of simulated treatment.

### Kinetics and kinematics of the molar and anchorage teeth

**Fig. 9:**
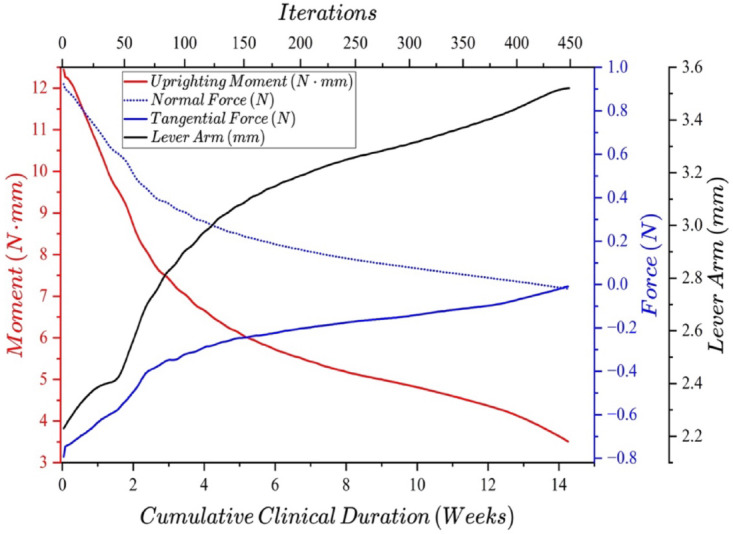
Evolution of the force system acting on the mandibular second molar during uprighting. The graph shows the uprighting moment in solid red, normal force in dotted blue, tangential force in solid blue, and lever arm length along the buccal tube slot in solid black throughout the iterative simulation. The x-axis is expressed in both iteration number (top) and equivalent clinical duration weeks (bottom)

The biomechanical results for the molar uprighting process when using a superelastic NiTi spring are expressed in terms of both clinical duration and iteration number. The evolution of the force system acting on the mandibular second molar throughout the simulation is shown in Fig. 9. The system was obtained from the reactive moments and forces computed for each iteration at the reference point representing the molar’s buccal tube to describe the overall load transfer from the superelastic NiTi spring. The extracted normal component corresponds to the vertical axis perpendicular to the inner surface of the slot (intrusive–extrusive direction), whereas the tangential component lies horizontal along the slot’s longitudinal axis (mesial–distal direction). The positive values are defined as extrusive and distal displacements, while negative values are defined as intrusive and mesial displacements. At the beginning of treatment, the spring generated a counter-clockwise uprighting moment of approximately 12.4 N·mm (~ 1265 g·mm), a normal extrusive force of 0.92 N (~ 94 g), and a tangential mesial force of about − 0.79 N (~ 81 g) acting on the molar. The lever arm length, defined here as the distance between the resultant upward mesial and downward distal contact forces at the opposing corners of the buccal tube slot, measured about 2.2 mm initially. As the molar approached its corrected position, all forces followed an almost comparable decreasing pattern. The moment gradually declined to about 3.5 N·mm (~ 357 g·mm) toward the end of the simulation. The normal force also decreased and eventually reversed direction, reaching a slight intrusive value of approximately − 0.02 N (~ 2 g), while the corresponding tangential mesial forces diminished progressively to almost negligible levels. Conversely, the lever arm length steadily increased to about 3.5 mm by the end of the simulation, reflecting a distal migration of the resultant contact force along the lower surface of the buccal tube slot. Additionally, as the molar attained its corrected angulation, the superelastic NiTi wire displayed some mesial sliding relative to the molar’s buccal tube as uprighting progressed.

**Fig. 10:**
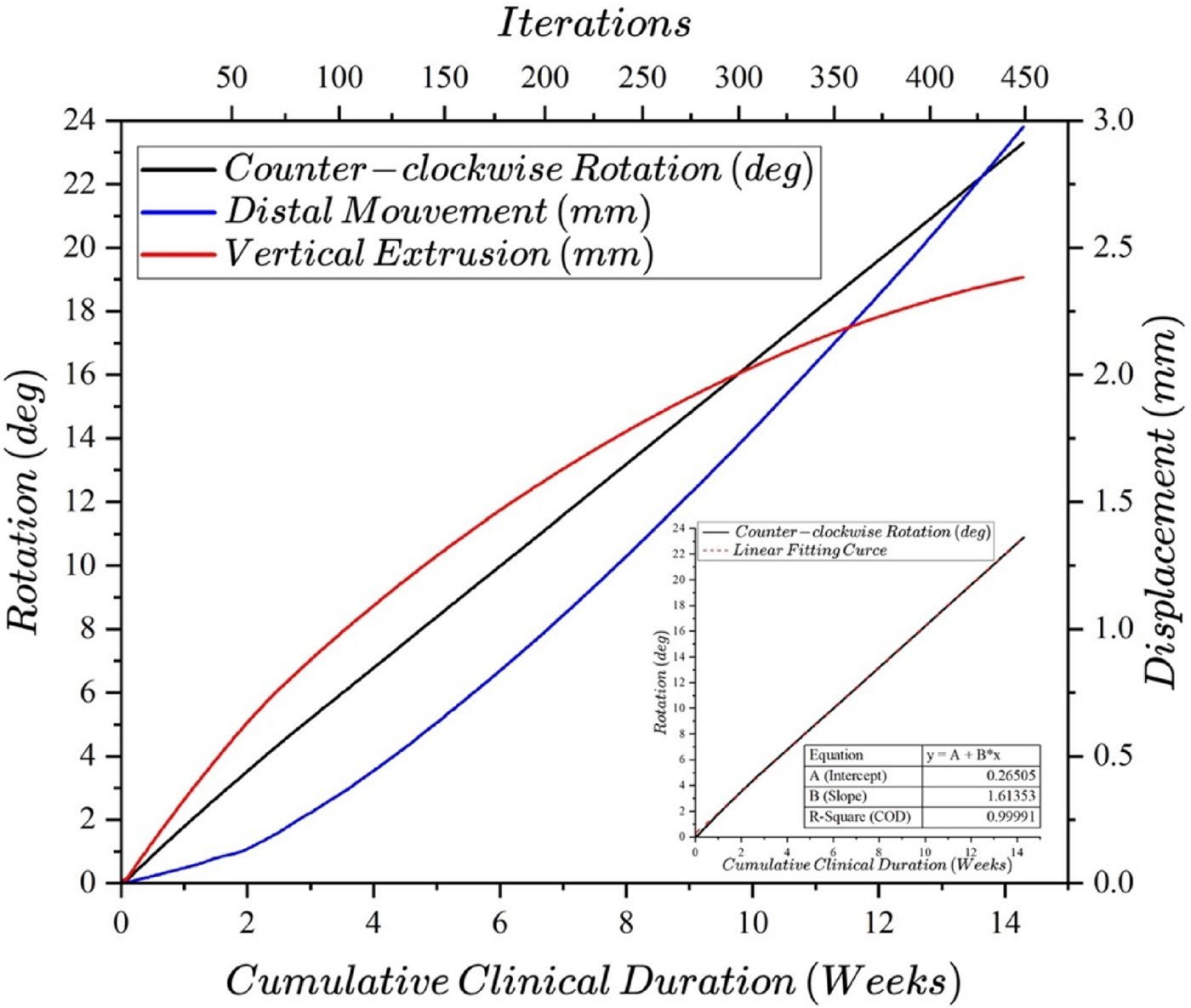
Changes in mandibular second molar rotation and displacement during the iterative simulation. The curves represent uprighting counter-clockwise rotation in black, normal extrusive displacement in red, and tangential distal displacement in blue over time. The x-axis is expressed in both iteration number (top) and equivalent clinical duration weeks (bottom). The inset within the plot area shows the linear fit of the uprighting rotation curve

Subsequent to this force system, Fig. 10 displays the changes in the mandibular second molar position throughout the uprighting treatment. The molar showed approximately 23.3° of counter-clockwise rotation over the simulated 14-week clinical duration, proceeding at an almost constant rate of 1.6° per week. This steady rotational behavior is confirmed by the high linear correlation between rotation and time (R² = 0.9998). In parallel, the molar showed about 2.4 mm of normal extrusive displacement and 3 mm of tangential distal displacement, both occurring gradually with the rotation. The highest mean stress inflicted on the molar’s PDL was approximately 20.3 KPa at the initial spring activation step. By the end of the simulation, this PDL stress was reduced to 13.3 KPa.

**Fig. 11:**
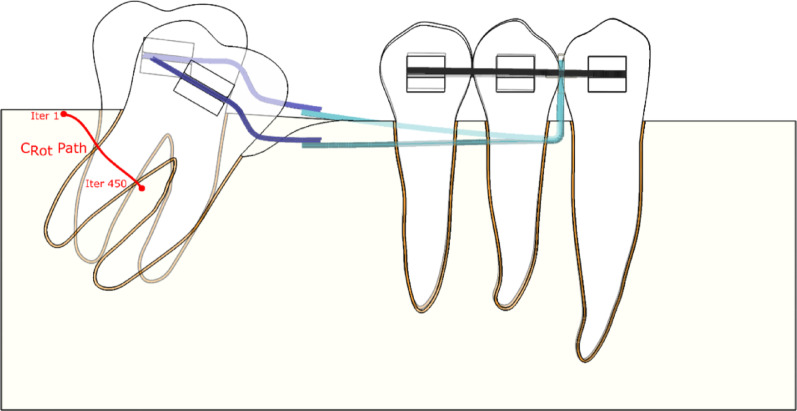
Superimposed configurations of all model components at the beginning and end of treatment simulation. The red path indicates the displacement of the mandibular second molar’s center of rotation (C_Rot_), showing its migration from an initial location outside the tooth’s boundary to a final position between the roots as uprighting progressed

An overall view of the movements produced by the superelastic NiTi uprighting spring during the treatment simulation is shown in Fig. 11. The figure presents the superimposed configurations of all model components at the beginning and end of the simulation. The red trajectory traces the displacement of the mandibular second molar’s center of rotation (C_Rot_), which was initially located outside the tooth’s boundary and thereafter progressively shifted between the roots by the end of treatment.

**Fig. 12:**
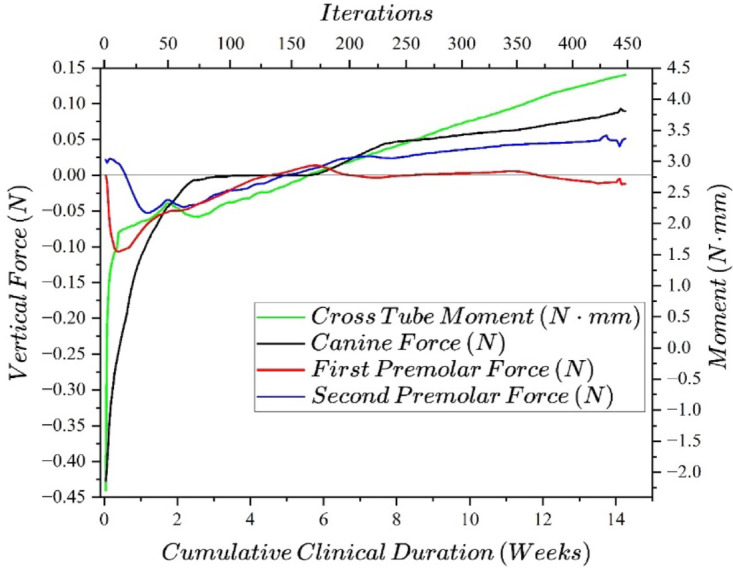
Evolution of the force system acting on the anchorage teeth during the iterative uprighting simulation. The green curve shows the moment generated by the cross tube on the anchorage segment, while the black, red, and blue curves represent the vertical forces transmitted respectively to the canine, first premolar, and second premolar. The x-axis is expressed in both iteration number (top) and equivalent clinical duration weeks (bottom)

The evolution of the force system acting on the anchorage teeth over the course of the simulation is shown in Fig. 12. The positive values are defined as counter-clockwise moment and extrusive displacement, while negative values are defined as clockwise moment and intrusive displacement. The cross tube connecting the SS component of the uprighting spring to the anchorage wire produced an overall counter-clockwise coupling moment on the anchorage segment. At the beginning of treatment, this moment was approximately − 2.3 N·mm (~ 235 g·mm) in the clockwise direction and displayed a sharp change during the initial activation phase. Within the first week, the moment direction reversed to reach nearly 2 N·mm (~ 203 g·mm) counter-clockwise. Thereafter, it continued to increase gradually, attaining about 4.4 N·mm (~ 450 g·mm) of counter-clockwise moment by the end of the simulation. Furthermore, the anchorage teeth were initially subjected to varying amounts of intrusive forces, with the canine showing the highest magnitude of about − 0.43 N (~ 44 g). From approximately week 5 onward, the vertical forces across all anchorage teeth became more uniform, displaying extrusive patterns. By the end of the simulation, a progressive and controlled redistribution of anchorage loads within the uprighting system was noted. The first premolar displayed a nearly neutral vertical load (~ 0 N), while the second premolar and canine showed about 0.05 N (~ 5 g) and 0.08 N (~ 8 g) of extrusive forces, respectively.

**Fig. 13:**
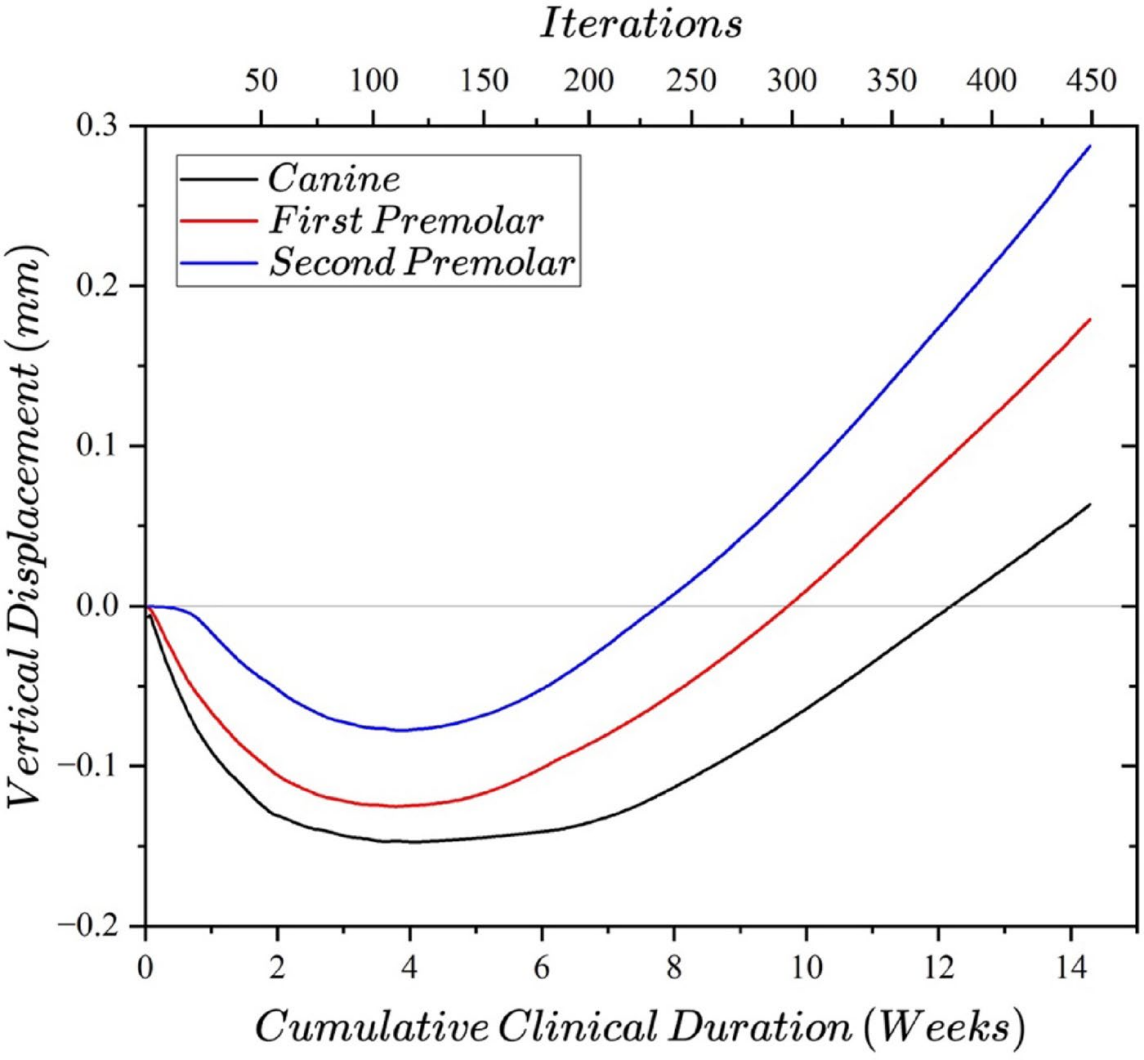
Vertical displacement of the anchorage teeth over the course of the iterative simulation. The black, red, and blue curves represent the vertical displacements of canine, first premolar, and second premolar, respectively. The negative values indicate intrusive displacements, and the positive values indicate extrusive displacements. The x-axis is expressed in both iteration number (top) and equivalent clinical duration weeks (bottom)

Figure 13 shows the changes in the canine, first premolar, and second premolar positions during the treatment simulation. During the early iterations, all anchorage teeth exhibited intrusive displacements. Around week 5, the direction of vertical displacement changed progressively to become extrusive, with the second premolar showing the largest final displacement. However, these movements resembled minimal values that would be difficult to detect clinically. The analyzed shift from intrusion to extrusion highlighted the changing load transfer within the cross tube and anchorage segment as the spring unloaded and force balance evolved.

Table 3 presents a summarized overview of the biomechanical effects of the superelastic NiTi uprighting spring from the FE simulation results, reported at 2-week clinical intervals. These data offer a simplified clinical reference for anticipating force magnitudes and displacement rate of the mandibular second molar during treatment.

**Table 3:** Predicted progression of molar uprighting with superelastic NiTi spring over the simulated clinical duration

Clinical duration (Weeks)	Uprighting moment (*N*.mm)	Rotation angle (deg)	Normal force (*N*)	Normal displacement (mm)	Tangential force (*N*)	Tangential displacement (mm)
Week 0	12.44	0.0	0.92	0.0	-0.79	0.0
Week 2	8.71	3.5	0.51	0.6	-0.49	0.1
Week 4	6.66	6.8	0.29	1.1	-0.29	0.4
Week 6	5.72	10.0	0.19	1.5	-0.22	0.8
Week 8	5.17	13.2	0.12	1.8	-0.17	1.3
Week 10	4.81	16.4	0.07	2.0	-0.14	1.8
Week 12	4.36	19.6	0.03	2.2	-0.09	2.3
Week 14	3.50	23.3	-0.02	2.4	-0.01	3.0

The normal direction corresponds to intrusion–extrusion. The tangential direction corresponds to mesio–distal. The positive values indicate extrusive and distal displacements. The negative values indicate intrusive and mesial displacements

## Discussion

### Validation of the EIS model and consistency of material behavior

Capturing the true mechanical response of superelastic NiTi alloy during orthodontic treatment is of utmost importance for achieving clinically reliable results [13, 31, 39]. Thus, the current study utilized an EIS model to provide an accurate representation of the material behavior when using FE analysis.

**Fig. 14:**
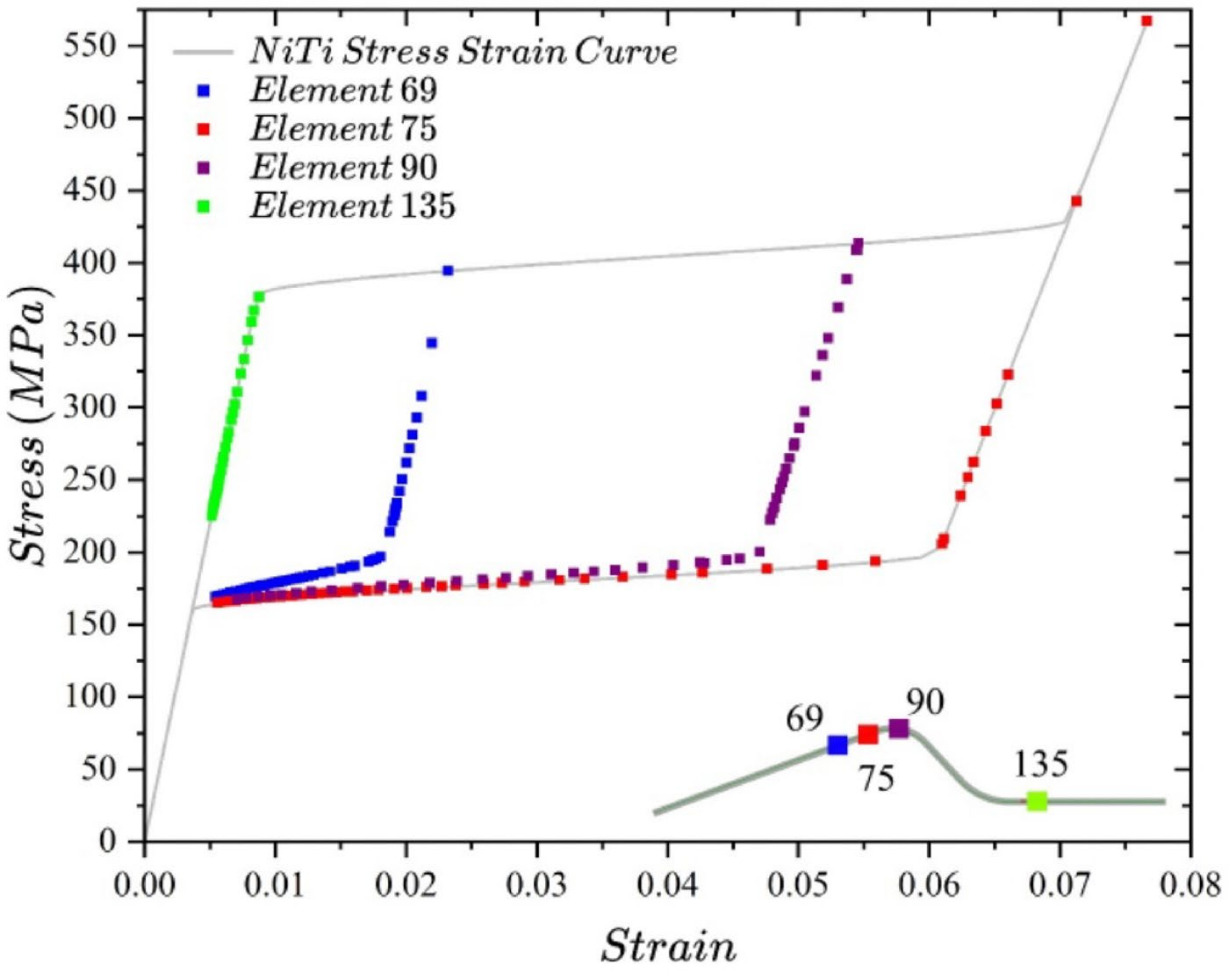
Element specific stress–strain responses of the spring throughout the iterative process of molar uprighting. The selected elements 69, 75, 90, and 135 represent distinct regions along the wire and exhibit localized transformation behaviors. To maintain visual clarity, only one out of every ten iterations was plotted, while the overall trend followed the characteristic superelastic NiTi stress–strain curve shown in grey

The element specific stress–strain responses shown in Fig. 14 confirm the robustness of the EIS formulation in reproducing the localized mechanical behavior of superelastic NiTi material during the iterative molar uprighting simulation. All element responses perfectly followed the referenced material stress–strain curve shown in gray color, from the initial spring activation step until completion of treatment. This demonstrates the model’s ability to preserve consistency of material’s behavior during both loading and unloading.

Four representative elements were selected to illustrate different deformation histories along the spring; these include elements 69, 75, 90, and 135. The results show that element 135 remained fully austenitic. Element 69 underwent partial transformation, while element 90 showed a broader transformation range. Element 75, which was located in the most deflected region, completed the martensitic transformation surpassing the plateau, and continued along the elastic martensitic slope of the superelastic NiTi curve. These findings indicate that the EIS model was able to accurately present the stress–strain state of each element at every iteration, showing progressive stress decrease along the expected unloading path. Thus, confirming the method’s capability to reproduce the nonlinear, path-dependent behavior of superelastic NiTi material, and overcoming the limitations of conventional iterative FE approaches.

The model also reproduced the stress range associated with stress-induced martensitic transformation in superelastic NiTi alloy. As shown in Fig. 14, the element-level stress and strain responses aligned well with the characteristic superelastic stress-strain curve, with transformation initiating at approximately 377 MPa and extending up to 567 MPa at its upper limit. This wider range reflects different stress distributions within the wire and the progressive engagement of elements undergoing transformation. These values are in agreement with those reported by previous literature [9, 12]. Furthermore, Gannoun et al. (2020) and Naceur et al. (2014) both conducted numerical studies that reported an almost similar range, where the martensitic fractions were found at a stress range of ~ 380 MPa and ~ 500 MPa within the superelastic NiTi wire [13, 39]. The current results confirm that the EIS approach successfully captured the response of superelastic NiTi during orthodontic loading and unloading while reproducing the progressive force redistribution characteristic of the uprighting spring in a continuous iterative process. This presents new opportunities for future analysis of different materials behavior under mechanical loadings.

### Force system and load transfer mechanics

The achieved results confirm that the superelastic NiTi uprighting spring’s effects present a two-couple force system, facilitated by the cross tube acting as a bracket engaging the anchorage wire [11]. The initial molar uprighting moment was ~ 12.4 N·mm (~ 1265 g·mm), and normal extrusive force 0.92 N (~ 94 g), both of which fall within previously reported ranges for molar uprighting appliances [2, 17, 26, 27, 49].

The initial clockwise coupling moment produced on the anchorage segment led to a predominant intrusive effect, particularly on the canine. This system configuration produced overall opposite acting moments and vertical forces; a counter-clockwise moment with extrusion on the molar and a clockwise moment with intrusion on the anchorage teeth, resembling the effects of Burstone’s class V geometry at initial stages of the treatment. However, as the uprighting progressed and load transfer changed during spring unloading, the anchorage segment’s moment and vertical force patterns became reversed, following the direction of the molar. A counter-clockwise moment was produced along with minor extrusion on the anchorage teeth. This overall shift in force dynamics produced similar effects to Burstone’s class III geometry by the end of the treatment simulation. The current findings indicate that more than one force system class can be produced by the superelastic NiTi uprighting spring [50].

**Fig. 15:**
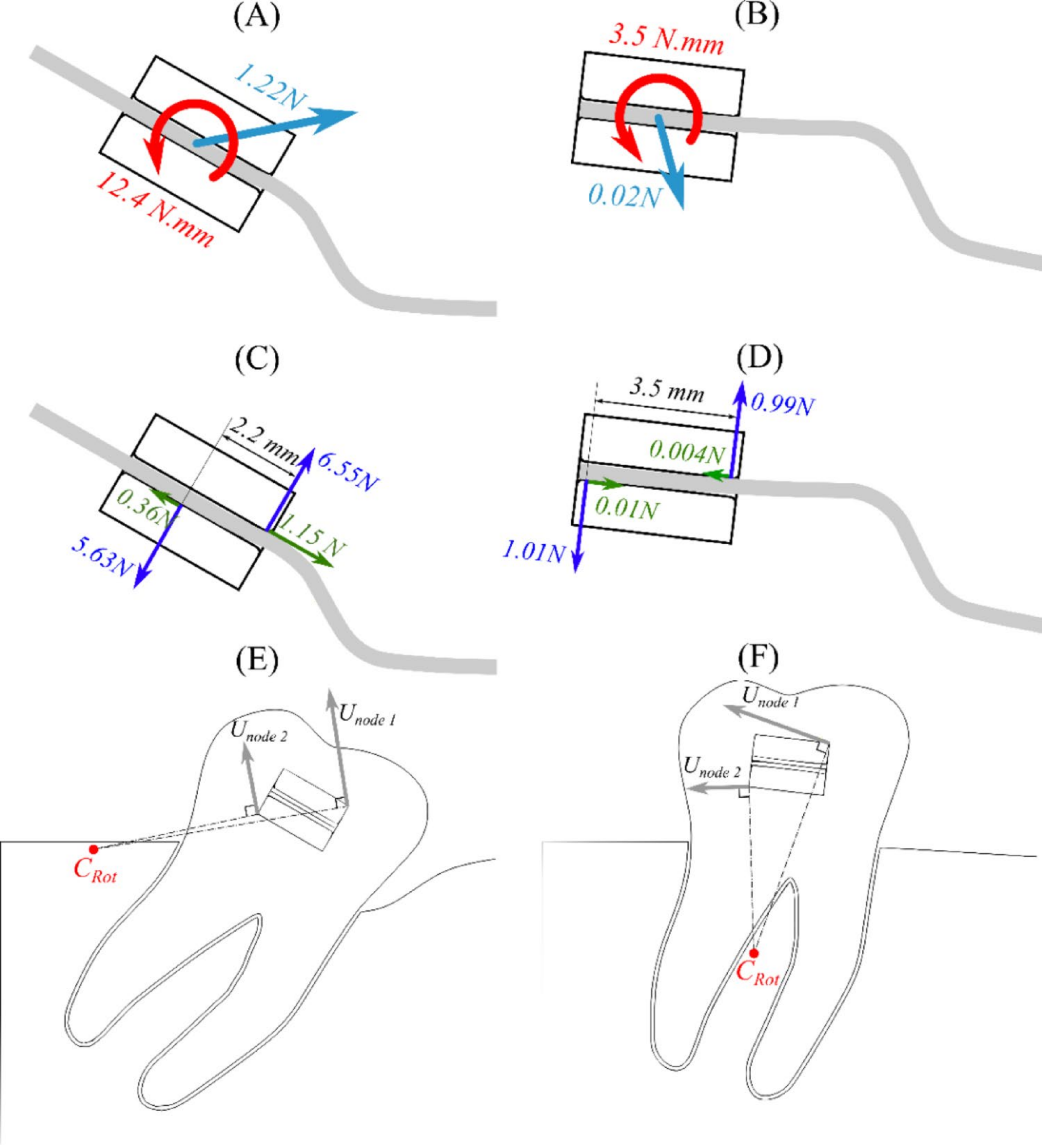
A detailed view of force system effects within the buccal tube slot during uprighting process. The load transfer mechanics and molar kinematics are shown at the initial spring activation step within the first iteration, and at the last iteration when treatment was completed. **A**,** B** Equal and opposite force and moment to the reactive values extracted at the buccal tube reference point, representing the actual load applied to the molar by the uprighting spring. **C**, **D** Contact-based normal and tangential force components (blue and green arrows respectively) acting on the buccal tube, showing the lever arm length responsible for the uprighting moment. **E**, **F** Nodal displacement vectors on selected buccal tube nodes illustrating how the molar’s C_Rot_ is determined and how it shifts from a disto-coronal to a more mesio-apical position as uprighting progressed

Additionally, the force system effects within the buccal tube slot, as well as molar kinematics at the beginning and end of treatment simulation, are further highlighted with a detailed view in Fig. 15. Insets (A) and (B) illustrate the reactive force and moment acting on the buccal tube at the first and last iterations, respectively. The load transfer mechanics are further broken down in insets (C) and (D), which show the contact forces between the superelastic NiTi spring and the buccal tube slot at the same two iteration stages. The normal resultant force, gained from the contacts on upper mesial and lower distal corners of the slot, as represented by the blue arrows, governs the overall extrusive and intrusive mechanics. In contrast, the tangential component of the force, represented by the green arrows, arises from the relative sliding of the buccal tube over the superelastic NiTi wire. The spatial offset between these forces defines the lever arm length used for moment interpretation. As uprighting progressed, this lever arm length gradually increased, indicating a distal migration of the resultant contact forces along the slot, further precipitated by mesial sliding of the spring wire relative to the buccal tube. This behavior reflected the evolving contact force distribution as the spring relaxes and returns to its original configuration before activation, subsequent to the inherent shape memory property of the superelastic NiTi material [11].

The molar’s C_Rot_ shifted from a disto-coronal position to a more mesio-apical one, as determined from the nodal displacements of the nodes on the buccal tube corners, which can be illustrated in insets (E) and (F) at the first and last iterations, respectively. This spatial migration reflects the progressive change in the line of action of the resultant force relative to the tooth’s center of resistance. Consequently, the moment-to-force ratio developed toward net extrusion on both the molar and anchorage segments, which might be accentuated due to the absence of opposing dentition in the current FE model. Additionally, the initial distal location of C_Rot_ led to a substantial amount of molar distalization during the uprighting process, a common finding for such scenarios [51]. This indicates a possible need for molar ligation in cases where this movement is not a prerequisite. Ligating the molar to the anchorage segment can possibly prevent spring wire sliding as well as the distal crown displacement, and instead produce a mesial root torque that uprights the molar [18].

Furthermore, the brief increase in iteration duration observed between iterations 50 and 75 (Fig. 6), likely resulted from temporary geometric and material reconfiguration within the superelastic NiTi and tooth system while martensitic transformation was still progressing on the loading plateau. Local rearrangement of the transformation front and transient redistribution of wire–bracket contact forces reduced the mean PDL stress, producing the observed dip before steady progression resumed. Beyond approximately 220 iterations, most of the superelastic NiTi elements had entered the unloading plateau, as indicated by the nearly horizontal trend in iteration duration, marking a quasi-steady mechanical equilibrium and sustained uprighting. This is consistent with the clinical transition from initial force peaks to low constant force delivery by the superelastic material [52, 53].

At the system level, the simulated uprighting moment, normal and tangential forces, along with C_Rot_ migration, reproduced the multi-phase biomechanical characteristics of the superelastic NiTi spring: an initial phase dominated by opposing moments; a transitional phase characterized by mesial sliding of the superelastic NiTi spring and progressive force redistribution; and lastly, a steady phase governed by controlled uprighting and superelastic recovery.

### Clinical and biomechanical implications

The biological threshold is the maximum amount of orthodontic force that supporting oral tissues can tolerate. Exceeding this limit with excessive loads can produce negative ramifications [54]. Furthermore, when it comes to various molar uprighting appliances, they can innately produce some undesirable biomechanical effects, such as extrusion and uncontrolled tipping [55, 56]. Therefore, a thorough analysis is required to enhance clinical outcomes and mitigate these unwanted side effects during the uprighting process.

The counter-clockwise uprighting moment generated on the molar was ~ 12.4 N·mm (~ 1265 g·mm), which lies in accordance with previous literature, as the moment produced for molar uprighting should not exceed an average of 1500 g·mm [49]. The highest values recorded for normal forces generated on the molar and anchorage teeth were approximately 0.9 N (92 g) and − 0.43 N (~ 44 g), respectively, both of which were within the average force range for orthodontic tooth movement [8], and specifically molar uprighting [57, 58]. Moreover, a previous FE study measured the maximum force delivered by a superelastic NiTi wire to be around 0.82 N [59]. Another in-vitro study reported vertical forces up to 100 g were produced when using a superelastic NiTi wire [60]. These values are at a close estimate to the ones achieved in the present study.

In the current model, the mean stress within the molar’s PDL decreased gradually from approximately 20.3 KPa at the initial treatment stage to 13.3 KPa at the end of the simulation. These values remain within the physiologic ranges for bone remodeling [29, 30, 61, 62]. Both values occurred on the mesio-apical side of the second molar’s distal root, where bone remodeling is typically initiated. This correspondence reflects the well documented relationship between the magnitude of periodontal stress and the biological rate of tooth movement [36].

The observed 2.4 mm of normal extrusive displacement in the molar is considered clinically relevant, as each millimeter of posterior extrusion can lead to 1.5–2.5 mm of anterior bite opening [63]. This can be attributed to a stronger mesial-upward force vector on the buccal tube corner, which also influenced C_Rot_ migration and the uprighting trajectory (Fig. 11). Therefore, the use of superelastic NiTi uprighting spring might require some clinical adjustments to reduce such unwanted biomechanical effects. A recommended approach would be to incorporate a 135° intrusion bend on the SS component of the spring. This would provide control over vertical dimensions, particularly in open bite cases [2].

The simulated clinical duration of approximately 14 weeks for molar uprighting is consistent with clinical research, as the length of molar uprighting treatment can range from 2 to 5 months on average, governed by multiple factors, among which are the degree of molar tilt and the type of orthodontic appliance used [1, 55, 64]. Furthermore, the current findings align with average treatment periods reported for comparable uprighting mechanics employing light and continuous forces [17, 27].

Roig-Vanaclocha et al. (2021) presented a case series with an average improvement of 24.5° in molar tilt over 3 months. These results lie in closeness to the ones achieved in the current study [64]. Another case study was able to correct a 30° tilted mandibular second molar in a period of 3 months, while utilizing a square 0.016 × 0.016-inch NiTi archwire for uprighting [65]. Two other studies used 0.016 × 0.022-inch superelastic NiTi archwires for uprighting mandibular tilted molars, which was completed within 3–4 months in both studies [66, 67]. Additionally, another case report utilizing the same uprighting spring as the current study, showed completed uprighting of a tilted mandibular second molar in a clinical period of 4 months [17]. This solidifies the closeness of current FE model findings to previous literature. Overall, the EIS-based framework was able to reproduce the kinetic and kinematic effects of the superelastic NiTi spring on uprighting a mesially tilted mandibular second molar while predicting clinically realistic outcomes. Hence, it bridges computational accuracy with clinical applicability, offering a validated tool for optimizing such spring mechanics in future designs.

In the current study, the superelastic NiTi uprighting spring achieved a 23.3° molar tilt correction in a clinical duration of 14 weeks at a rate of 1.6° per week, with a PDL stress range of 20.3–13.3 KPa. According to AlKahlan et al. (2025), the conventional molar uprighting spring achieved 22° of molar uprighting in a clinical duration of 12 weeks at a rate of 1.83° per week, with a PDL stress range of 28–3.5 KPa. This indicates that although the uprighting was slower for the superelastic NiTi spring, it achieved molar correction at a more biologically abiding rate, highlighting its superiority in delivering low constant forces. The current spring also produced less distal but slightly higher extrusive molar displacement in comparison to a conventional uprighting spring [36].

Furthermore, the conventional uprighting spring provides a force system that can be tailored by the operator; yet, the possibility of delivering higher forces at a more rapid rate remains to be warranted. Careful observation of forces and tooth movements during treatment progression is still required, with a possible need for reactivation during follow-up appointments in order to achieve the desired molar uprighting [6, 7]. Nevertheless, the superelastic NiTi uprighting spring provides a more efficient and biologically desired force system that delivers continuous low forces, with reduced reactivation need [2, 17, 18].

### Limitations and future work

The present study is subject to certain limitations inherent to the adopted modeling approach. The analysis was based on a 2D planar simplification, which, while computationally efficient, does not capture the bucco-lingual responses and associated out of plane load transfer that occur in-vivo. Nevertheless, the 2D formulation remains an effective and validated approach for depicting the primary load transfer patterns and tooth displacement mechanics during molar uprighting [36, 68, 69]. The model assumed bonded contact between the bone–PDL–root surfaces, and frictional contact between the superelastic NiTi spring and the buccal tube slot. Although these model assumptions are realistic, they may not fully represent the variability of interfacial mechanics observed clinically. In addition, patient specific anatomical variations were not considered, and the PDL thickness was assumed uniform, which neglects natural geometric variability that could influence local stress concentrations and tooth movement. The iterative geometric update of the bone, PDLs, and teeth was considered to represent the computational outcome of the cumulative remodeling process at each step, rather than an explicit implementation of a mechanobiological bone-remodeling algorithm. This modeling strategy was consistent with the main objective of the study, which was to evaluate the proposed EIS material model for superelastic NiTi and apply the previously validated clinical time-estimation method [36]. Nevertheless, this approach remains an idealization, as orthodontic bone remodeling is time-dependent, spatially heterogeneous, and can be represented computationally using different remodeling algorithms and assumptions. Moreover, the absence of occlusal contacts with opposing dentition limits the ability to reproduce vertical equilibrium similar to in-vivo conditions in order to predict the subsequent anchorage stability.

Future research might benefit from extending the current study to a 3D model analysis, providing deeper insights into the biomechanical impact of superelastic NiTi uprighting springs, particularly in the bucco-lingual direction. The incorporation of intrusion and extrusion bends in the SS component of the spring, and evaluating their influence on the force system, would offer valuable information that optimizes uprighting mechanics. Furthermore, exploring the mechanical play between the SS and NiTi spring junctions, such as increasing the junction length, could reveal strategies to enhance the stress-induced martensitic transformation and overall spring efficiency. Additionally, integrating opposing dentition and PDL with variable thickness into the simulation, as well as accounting for different bone types and mechanobiological laws for remodeling, would improve the realism and clinical translatability of the outcomes. Lastly, analyzing and comparing different molar uprighting appliances, such as miniscrew-supported springs, could enhance the overall understanding and effectiveness of treatment modalities.

## Conclusions

A vital aspect of improving orthodontic treatment outcomes resides in understanding the biomechanics behind molar uprighting springs. This FE study introduced an EIS modeling framework capable of reproducing the kinetic and kinematic behavior of a superelastic NiTi molar uprighting spring. The model achieved 23.3° of molar uprighting, 3 mm of distal displacement, and 2.4 mm of extrusive displacement over a 14-week span of equivalent clinical duration. The model successfully tracked the alloy’s stress-induced martensitic transformation, capturing its spatial and temporal evolution during spring activation and molar uprighting. By incorporating the nonlinear and path-dependent properties of superelastic NiTi, the EIS approach yielded clinically representative predictions consistent with physiological tooth movement. These findings establish a validated computational basis for optimizing NiTi-based orthodontic appliances, provide a foundation for future evaluation of different elastic materials’ behavior using FE analysis, and pave a path for novel advancements in appliance design that enhance treatment efficiency and patient care.

## Data Availability

All data generated or analysed during this study are included in this published article.

## Abbreviations

2D: Two-dimensional
3D: Three-dimensional
CAD: Computer-assisted design
C_Rot_: Center of rotation
CT: Computed tomography
EIS: Element-iteration-specific
FE: Finite element
NiTi: Nickel Titanium
PDL: Periodontal ligament
SS: Stainless steel
